# Supplementary figures and images for: Overexpression of KIAA1199 is an independent prognostic marker in laryngeal squamous cell carcinoma
Source: PeerJ. 2020 Sep 7;8:e9637. doi: 10.7717/peerj.9637 (PMC7482636; doi:10.7717/peerj.9637)

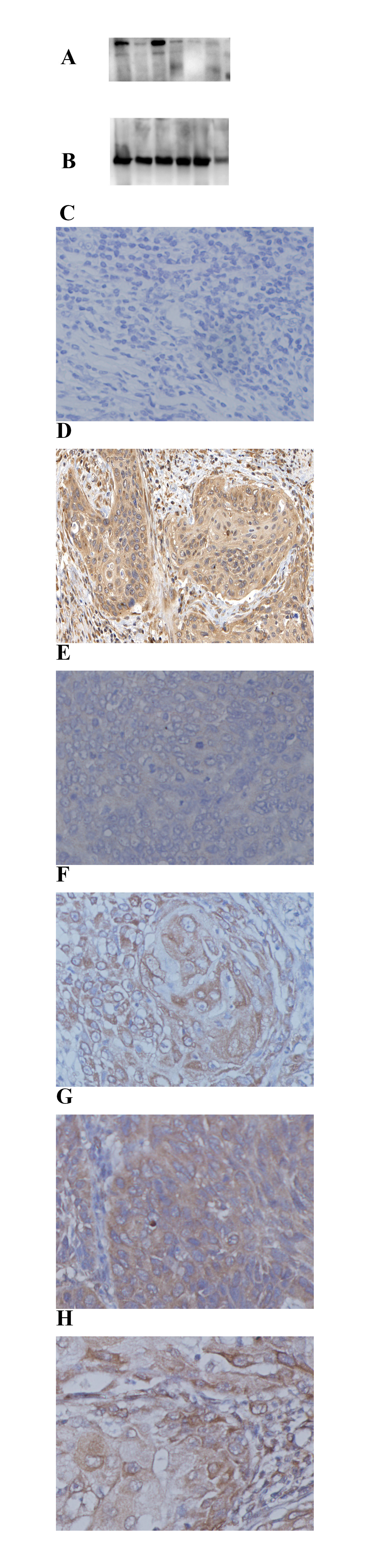

Supplement: Supplemental Information 1 — (A) The raw gel of Western blotting for KIAA1199 and (B) The raw gel of Western blotting for GAPDH. Figures (C)–(H) are IHC raw photos. [file peerj-08-9637-s001.png]
